# STAT3-induced lncRNA HAGLROS overexpression contributes to the malignant progression of gastric cancer cells via mTOR signal-mediated inhibition of autophagy

**DOI:** 10.1186/s12943-017-0756-y

**Published:** 2018-01-12

**Authors:** Jin-Fei Chen, Peng Wu, Rui Xia, Jian Yang, Xin-Ying Huo, Dong-Ying Gu, Cui-Ju Tang, Wei De, Fen Yang

**Affiliations:** 1 Department of Oncology, Nanjing First Hospital, Nanjing Medical University, Nanjing, People’s Republic of China; 2 Department of Clinical Laboratory, Nanjing Chest Hospital, Nanjing, People’s Republic of China; 3 Department of Biochemistry and Molecular Biology, School of Basic Medical Sciences, Nanjing Medical University, Nanjing, 211166 People’s Republic of China

**Keywords:** lncRNA, HAGLROS, miR-100-5p, mTOR, Gastric cancer, Autophagy

## Abstract

**Background:**

Long noncoding RNAs (lncRNAs) are an important class of functional regulators involved in human cancers development, including gastric cancer (GC). Studying aberrantly expressed lncRNAs may provide us with new insights into the occurrence and development of gastric cancer by acting as oncogenes or tumor suppressors. In this study, we aim to examine the expression pattern of lncRNA HAGLROS in GC and its clinical significance as well as its biological role in tumor progression.

**Methods:**

Bioinformatics analysis and qRT-PCR were performed to detect the relative expression of HAGLROS in GC tissues and cell lines. Gain or loss of function approaches were used to investigate the biological functions of HAGLROS. The effect of HAGLROS on proliferation was evaluated by MTT, colony formation assay and nude mouse xenograft model. Wound healing and Transwell assays were used to study the invasion and migration of GC cells. FISH, RIP, RNA-seq, Luciferase report assays, RNA pulldown and Western blot were fulfilled to measure molecular mechanisms. Results are shown as means ± S.D. and differences were tested for significance using Student’s t-test (two-tailed).

**Results:**

We screened out HAGLROS, whose expression was significantly increased and correlated with outcomes of GC patients by publicly available lncRNAs expression profiling and integrating analyses. Exogenous down-regulation of HAGLROS expression significantly suppressed the cell proliferation, invasion and migration. Mechanistic investigations showed that HAGLROS was a direct target of transcriptional factor STAT3. Moreover, HAGLROS knockdown decreased mTOR expression and increased autophagy-related genes ATG9A and ATG9B expression. Further investigation showed that HAGLROS regulated mTOR signals in two manners. In the one hand, HAGLROS competitively sponged miR-100-5p to increase mTOR expression by antagonizing miR-100-5p-mediated mTOR mRNA inhibition. On the other hand, HAGLROS interacted with mTORC1 components to activate mTORC1 signaling pathway which was known to be an important negative signal of autophagy. Here activation of mTORC1 signaling pathway by HAGLROS inhibited autophagy, thereby promoted excessive proliferation and maintained the malignant phenotype of GC cells.

**Conclusion:**

The present study demonstrates that HAGLROS overexpression contributes to GC development and poor prognosis and will be a target for GC therapy and further develop as a potential prognostic biomarker.

**Electronic supplementary material:**

The online version of this article (10.1186/s12943-017-0756-y) contains supplementary material, which is available to authorized users.

## Background

Recent evidence indicates that although more than 70% of the eukaryotic genome is transcribed, only approximately 1 to 2% of the transcriptome contributes to protein-coding RNA, suggesting that transcription is not limited to the protein-coding portion of the eukaryotic genome but includes other non-protein-coding sections [1, 2]. Based on their sizes, these transcribed noncoding RNAs (ncRNAs) can be classified as small, medium and long. Small ncRNAs range from 18 to 31 bp in length, whereas long ncRNAs range in size from 200 bp to over several hundred kilobases. Medium ncRNAs measure between 31 bp and 200 bp and contain mainly snRNAs and snoRNAs. Long noncoding RNAs (lncRNAs) are of interest because emerging evidence indicates that a subset of long noncoding RNAs mediate their biological functions using chromatin as a substrate to interact with the genetic information encoded in the genome [3].

Gastric cancer (GC) is ranked the fifth most common malignant neoplasm in the world, with approximately 951,600 new diagnoses and 723,100 deaths in 2012 [4]. Despite the decreased mortality rate of GC in recent years, it is still the second most common cause of cancer death [5, 6]. Further exploration of the molecular mechanisms underlying GC occurrence and development is urgently needed. Studying the aberrantly expressed lncRNAs involved in signaling pathways in GC may provide us with new insights into the occurrence and development of this disease. By acting as oncogenes or tumor suppressors, lncRNAs contribute to GC occurrence and development. Several lncRNAs, such as HULC, MALAT1, lncRNA-ATB and HOTTIP, have been demonstrated oncogenic activity [7–11], while other lncRNAs, including HOTAIR, GAS5 and PTENP1, are considered tumor suppressors [12–14]. The cellular localization of lncRNAs is varied. Majority of lncRNAs are localized to the nucleus (MALAT1 and NEAT1), some are distinctively found in the cytoplasm (DANCR and OIP5-AS1), and certain lncRNAs are found in both locations (TUG1, CasC7 and HOTAIR) [15]. The cellular localization of lncRNAs is intended for indulging in range of physiological activities from chromatin remodeling to translational regulation [16]. The basic structural and interactive capabilities of lncRNAs with other cellular biomolecules can help distinguish and specifically reveal their central roles in tumorigenesis. LncRNA–DNA, lncRNA–RNA and lncRNA–protein interactions are especially important. lncRNAs are involved in various levels of regulation, including transcriptional repression by binding to the PRC2 (Polycomb Repressive Complex 2) [12]. LncRNAs can also serve as a ‘sponge’ to titrate miRNAs, thus participating in post-transcriptional processing [17, 18]. The most important biomolecular interactions of lncRNAs are with RNA-binding proteins. All the classic molecular mechanisms of lncRNAs, such as guiding, scaffolding and decoying, are ultimately executed through interactions with proteins [19, 20].

The mammalian target of rapamycin (mTOR) signaling pathway integrates both intracellular and extracellular signals and functions as a central regulator of cell metabolism, growth, proliferation and survival. Activation of the mTOR pathway has a substantial regulatory role in cell proliferation and cell cycle progression [21]. The mTOR protein forms at least two distinct multiprotein structures: mTOR complex 1 (mTORC1) and 2 (mTORC2). These complexes share the catalytic mTOR subunit, mammalian lethal with sec-13 protein 8 (mLST8, also known as GbL), the DEP domain containing mTOR-interacting protein (DEPTOR), and the Tti1/Tel2 complex. Regulatory-associated protein of mammalian target of rapamycin (raptor) and proline-rich Akt substrate 40 kDa (PRAS40) are specific to the mTORC1 complex, whereas rapamycin-insensitive companion of mTOR (rictor), mammalian stress-activated map kinase-interacting protein 1 (mSin1) and protein observed with rictor 1 and 2 (protor1/2) are only part of the mTORC2 complex [21].

Here, we report the characterization of HAGLROS as an lncRNA highly expressed in GC and implicated in the regulation of cell proliferation and migration. In the present study, we identified HAGLROS by analyzing publicly available lncRNA expression profiling data from GC. HAGLROS has only one transcript, a 699 bp lncRNA, according the NCBI (NCBI Reference Sequence: NR_110457.1). HAGLROS was up-regulated in GC tissues and served as an independent predictor for overall survival. In addition, HAGLROS was a direct transcriptional target of STAT3, and HAGLROS regulated GC cell proliferation both in vitro and in vivo. Mechanistic studies showed that HAGLROS regulated mTOR signaling by functioning as a competing endogenous RNA (ceRNA), which suppressed the degradation of mTOR mRNA by competing with miR-100-5p. HAGLROS also functioned as an mTORC1 binding partner, interacting with mTOR, Raptor and PRAS40 and stabilizing the complex. Activated mTOR promoted excessive proliferation and maintained the malignant phenotype of GC cells by inhibiting autophagy. Taken together, our study revealed that STAT3-induced lncRNA HAGLROS overexpression contributes to the malignant proliferation and invasion of GC cells via mTOR signal-mediated inhibition of autophagy and predicts poor outcomes in GC patients. These results provide important experimental evidence for the diagnosis and treatment of GC and suggest that HAGLROS may serve as a target for new therapies in human GC.

## Methods

### Microarray analysis

GC gene expression data were downloaded from The Cancer Genome Atlas (TCGA) and National Center for Biotechnology Information (NCBI) Gene Expression Omnibus (GEO) dataset. Heat maps representing differentially regulated genes were generated using Cluster 3.0 software (http://hemi.biocuckoo.org). Microarray data have been deposited under accession number GSE58828.

### Cell lines

The human gastric cancer cell lines SGC-7901, BGC-823, HGC-27, MGC-803 and AGS and the normal gastric epithelium cell line (GES1) were obtained from the Chinese Academy of Sciences Committee on Type Culture Collection Cell Bank (Shanghai, China). They are cultivated in Dulbecco’s modified Eagle’s medium (DMEM) supplemented with 10% fetal bovine serum (FBS) in a humidified atmosphere containing 5% CO_2_ at 37 °C.

### Study subjects

We obtained 84 paired GC and adjacent non-cancerous tissues from patients who underwent surgery at Nanjing First Hospital of Jiangsu Province in China between 2011 and 2012 and who were diagnosed with GC based on histopathologic evaluation. No local or systemic treatment was conducted in these patients before surgery. All collected tissue samples were preserved in RNA Transport (OMEGA Engineering Inc., Norwalk, CT, USA) and immediately frozen at −80 °C until required. The clinical characteristics of all patients are listed in Additional file 1: Table S1.

### Antibodies and reagents

The following antibodies were used in the study: mTOR Pathway Antibody Sampler Kit, anti-P70S6K, anti-p-P70S6K, anti-LC3, anti-P62 and secondary antibodies were purchased from Cell Signaling Technology (Danvers, MA, USA). 3-MA was purchased from Sigma-Aldrich (St. Louis, MO, USA).

### qRT-PCR analysis

qRT-PCR was used to detect expression levels of HAGLROS and other genes in GC tissues and cells following the manufacturer’s instructions (LightCycler® 480, Roche, Basel, Switzerland). GAPDH and β-actin were used as controls. Essential details referred to the MIQE guidelines [22]. Primers are listed in Additional file 2: Table S2. Total RNA was extracted using Trizol (Invitrogen, Grand Island, NY, USA), according the manufacturer’s instructions. cDNA was synthesized using PrimeScript™ RT reagent Kit (Takara Bio USA, Inc., Mountain View, CA, USA, No. RR047A).

For miRNA quantification, the Bulge-loop™ miRNA qRT-PCR Primer Sets (one RT primer and a pair of qRT-PCR primers for each set) specific for miR-100-5p is designed by RiboBio (Guangzhou, China). cDNA was synthesized using PrimeScript™ RT reagent Kit (Takara Bio USA, No. RR037A).

### Plasmid construction and cell transfection

The full-length complementary cDNA of human HAGLROS was synthesized by Invitrogen and cloned into the expression vector pc-DNA3.1 (Takara Bio USA, Inc.) the small hairpin RNA (shRNA) of the HAGLROS was provided by Invitrogen Corporation (Grand Island, NY, USA), and the final construct was verified by sequencing. Plasmid vectors for transfection were prepared using DNA Midiprep Kits (Qiagen, Hilden, Germany) and transfected into GC cells using Lipofectamine 2000 (Invitrogen). The siRNAs were transfected into GC cells using Lipofectamine 2000 according the manufacturer’s instructions. All siRNA and shRNA sequences are listed in Additional file 2: Table S2.

### Luciferase reporter assays

The STAT3-binding motif in the promoter region of HAGLROS was identified by JASPAR (http://jaspar.genereg.net/). The different fragment sequences were synthesized and then inserted into the pGL3-basic vector (OMEGA Engineering Inc.) and co-transfected with STAT3 plasmid into 293T cells. The miR-100-5p sequence was synthesized, inserted into the pGL3-basic vector and co-transfected with wild-type and mutant HAGLROS (the binding site for miR-100-5p was mutated) plasmid into 293T cells. The 3′-UTR of mTOR was cloned into the luciferase vector and transfected into 293T together with miR-100-5p mimics, the miR-100-5p inhibitor, the HAGLROS plasmid or the negative control. All vectors were verified by sequencing, and luciferase activities were assessed using a Dual Luciferase Assay Kit (OMEGA Engineering Inc.) in accordance with the manufacturer’s instructions.

### Cell proliferation

Cell proliferation ability was examined using a Cell Proliferation Reagent Kit I (MTT, Sigma-Aldrich). Absorbance values were measured at the wavelength of 490 nm. Inhibitory rates were calculated by Microsoft Excel (Microsoft Corporation, Redmond, WA, USA).

Colony formation assays were performed to monitor the cloning capability of GC cells. Cells were seeded in 6-well plates at 1 × 10^3^ cells/well and cultivated in DMEM (without any cytokine) with 10%FBS for 14 days, with the medium being replaced every 4 days. Colonies were fixed with methanol and stained with 0.1% crystal violet (Sigma-Aldrich) in PBS for 15 min. Colony formation was determined by counting the number of stained colonies.

### Cell migration and invasion

A wound healing assay was used to test for cell migration capabilities. A total of 2–4 × 10^5^ cells were seeded in 6-well plates, cultured for 12–24 h, and transfected with siRNAs or a control siRNA and with pc-DNA3.1-HAGLROS or a control vector. Once cultures reached 85% confluency, the cell layer was scratched with a sterile plastic tip, washed with culture medium, and then cultured for 24 h and 48 h. At different time points, images of the plates were acquired using a microscope (Olympus, Tokyo, Japan) and relative areas of wounds using Image J software to quantify and calculate the significance of the observed event.

For the invasion assays, 1 × 10^5^ cells in serum-free medium were placed into the upper chamber of an insert coated with Matrigel. Medium containing 10% FBS was added to the lower chamber. After incubation for 24 h, the cells remaining on the upper membrane were removed with cotton wool. Cells that had migrated or invaded through the membrane were fixed with methanol, stained with 0.1% crystal violet, imaged, and counted using an inverted microscope (Olympus).

### Establishment of xenografts and in vivo studies

Animal studies were performed in accordance with the criteria outlined in the ‘Guide for the Care and Use of Laboratory Animals’ prepared by the National Academy of Sciences and published by the National Institutes of Health (USA). Four-week-old female athymic BALB/c nude mice were maintained under specific pathogen-free conditions and manipulated according protocols approved by the Shanghai Medical Experimental Animal Care Commission and the Committee on the Ethics of Animal Experiments of the Nanjing Medical University. HAGLROS shRNA and Ctrl shRNA stably transfected SGC-7901 cells were harvested, and 1 × 10^7^ cells were subcutaneously injected into a single side of each mouse. Tumor sizes were measured by caliper and recorded every 3 days. The tumor volumes were calculated from the length (the longest diameter across the tumor) and width (the corresponding perpendicular diameter) using the following formula: π/6 × length × width^2^. After 20 days of growth, animals were killed, and tumors were resected and preserved at −80 °C or in formaldehyde for qRT-PCR and IHC staining, respectively.

### FISH and subcellular fractionation

FISH assay was performed using a Ribo™ Fluorescent In Situ Hybridization Kit and Ribo™ lncRNA FISH Probe Mix (Ribo, Guangzhou, China) according to the manufacturer’s protocols. The separation of nuclear and cytosolic fractions was performed using a PARIS Kit (Life Technologies, Carlsbad, CA, USA) according to the manufacturer’s instructions.

### RNA immunoprecipitation

An EZMagna RNA immunoprecipitation (RIP) Kit (Millipore, Bedford, MA, USA) was used following the manufacturer’s protocol. BGC-823 and SGC-7901 cells were lysed in complete RIP lysis buffer (containing proteinase inhibitor and phosphatase inhibitor), and the cell extract was incubated with magnetic beads conjugated with specific antibodies or control IgG for 6 h at 4 °C. Beads were washed and incubated with proteinase K to remove proteins. Finally, purified RNA was subjected to qRT-PCR analysis.

### RNA pull-down assay

RNAs were in vitro transcribed using T7 RNA polymerase (Ambion Inc., Austin, TX, USA), purified using an RNeasy Plus Mini Kit (Qiagen), and treated with RNase-free DNase I (Qiagen). Transcribed RNAs were biotin labeled with Biotin RNA Labeling Mix (Ambion Inc.). Positive, negative, and biotinylated RNAs were mixed and incubated with BGC-823 cell lysates. Magnetic beads were added to each binding reaction, followed by incubation at room temperature. Beads were washed with washing buffer, and eluted proteins were examined by Coomassie brilliant blue (Beyotime, Shanghai, China) staining and Western blot analysis.

### Chromatin immunoprecipitation

Chromatin immunoprecipitation (ChIP) experiments were performed using the MagnaChIP Kit (Millipore) according to the manufacturer’s instructions as described previously [23]. ChIP assay related primers are listed in Additional file 2: Table S2.

### Western blot analysis and immunoprecipitation

Western blot analysis and immunoprecipitation were performed according to standard protocols as described previously [24].

### LC3-II punctuation assay

For detecting the LC3-II punctuation, BGC-823 cells were transiently co-transfected with GFP-LC3 and HAGLROS siRNAs for 24 h and then seeded in a 24-well plate covered with 14 × 14 mm slips for next 24 h. After that, cells were fixed, permeabilized, and incubated with DAPI for 10 mins, and then adhered to coverslips after PBS washing. Cells on coverslips were observed using a confocal microscope (Olympus).

### Immunohistochemical (IHC) analysis

To quantify Ki67 expression, both the intensity and extent of immunoreactivity were evaluated and scored. IHC staining and score evaluation were performed according to standard protocols as described previously [25].

### Statistical analysis

Differences between groups were assessed by a paired, two tailed Student’s t-test. The Chi-square test was used to analyze the pathologic features of HAGLROS expression in GC. The survival curves are drawn using Kaplan-Meier survival plots and tested using log-rank tests. Univariate and multivariate Cox proportional hazards modeling was used to determine the effects of variables on survival. All statistical analyses were performed using SPSS 22.0 software (IBM SPSS, Chicago, IL, USA).

## Results

### Expression of HAGLROS is up-regulated in human GC tissues and correlates with poor prognosis

**Fig. 1:**
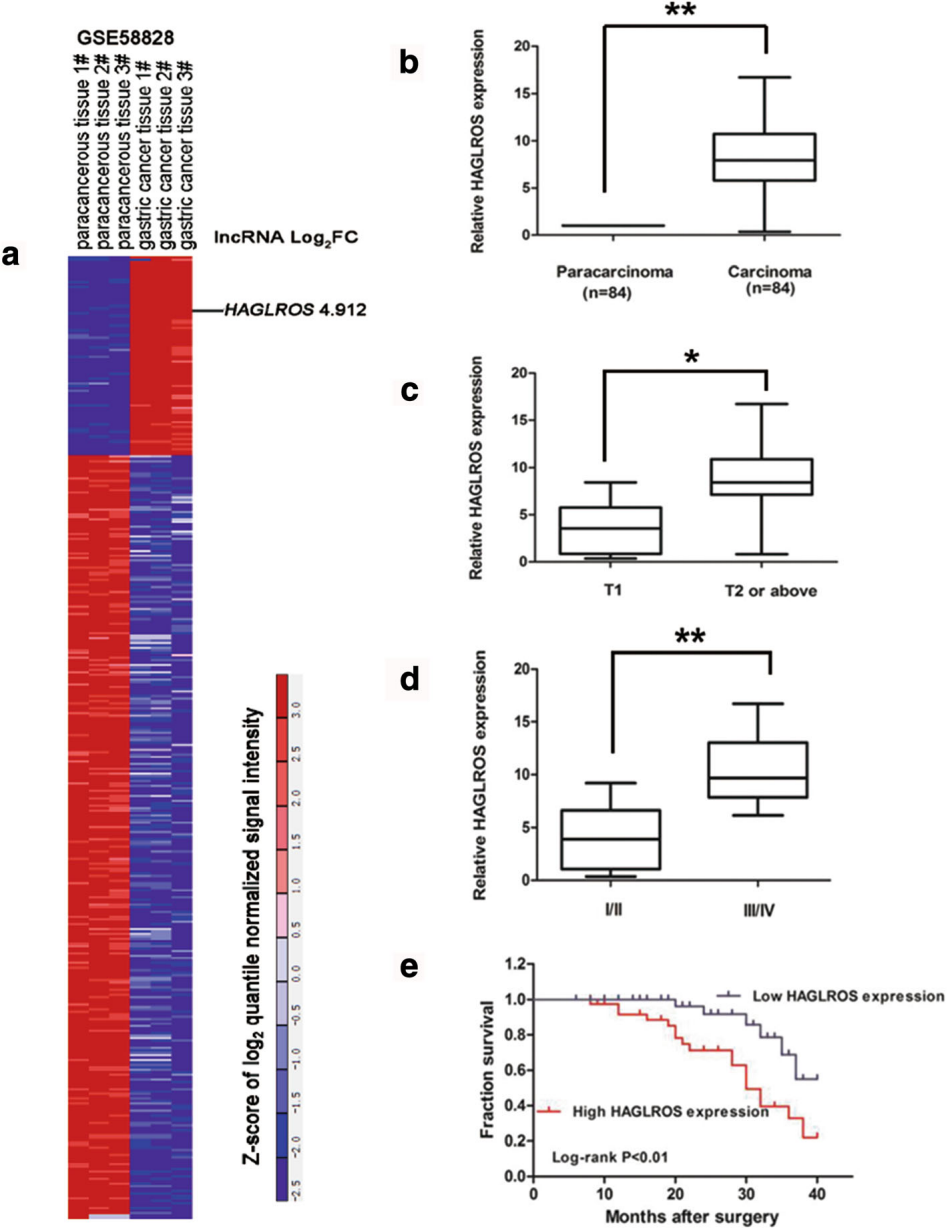
lncRNA HAGLROS is overexpressed in GC tissues and associated with the outcome of GC patients. **a** Hierarchical clustering analysis shows lncRNAs that were differentially expressed (fold change >2; *P* < 0.05) in GC and normal tissues from GEO datasets. **b** HAGLROS expression was analyzed by qRT-PCR in GC and adjacent non-tumor tissues (*n* = 84). The results are presented as the fold-change in tumor tissues relative to normal tissues. **c** and **d** Higher HAGLROS levels were positively correlated with advanced invasion depth and TNM stage. **e** Survival times of patients with high HAGLROS expression were decreased compared to those patients with low HAGLROS expression. *P < 0.05, **P < 0.01

From analysis of microarray profile GSE58828 of GEO dataset and mapsoft (http://lncrnamap.mbc.nctu.edu.tw/php/search.php), we identified differentially expressed 398 lncRNAs, including upregulated 82 lncRNAs (Fig. 1a). We selected the most differentially expressed 10 lncRNAs and validated their expression in 12 cases GC patient tissues. Results showed that HAGLROS is the most upregulated and well repeatedly expressed lncRNA (Additional file 3: Figure S1). Furthermore, we measured HAGLROS expression levels in tissue samples from 84 GC patients. qRT-PCR assays showed that HAGLROS expression was significantly higher in the cancer tissues than in the adjacent normal tissues (Fig. 1b). By analyzing clinic-pathological factors, we found that the high HAGLROS expression was correlated with poor prognosis in GC patients. There was an obvious positive correlation between higher HAGLROS levels and increased invasion depth and TNM stage (Fig. 1c and d). We divided samples into high (above the mean, *n* = 44) and low (below the mean, *n* = 40) HAGLROS expression groups for analysis based on the median value of HAGLROS levels. Clinic-pathological factors between the two groups are shown in Additional file 1: Table S1. HAGLROS levels were also correlated with tumor invasion depth and TNM stage. No relation was found between HAGLROS expression and other factors, e.g., sex, age or histological grade. As shown in Fig. 1e, elevated HAGLROS levels predicted a poor prognosis in patients with GC. Multivariate analysis further revealed that HAGLROS expression could be regarded as a potential diagnostic biomarker for overall survival in patients with GC (*P* = 0.005), for TNM stage (*P* = 0.019) and for lymph node metastasis (*P* = 0.001) (Additional file 4: Table S3).

### Elevated HAGLROS expression is associated with GC cell proliferation and invasion

**Fig. 2:**
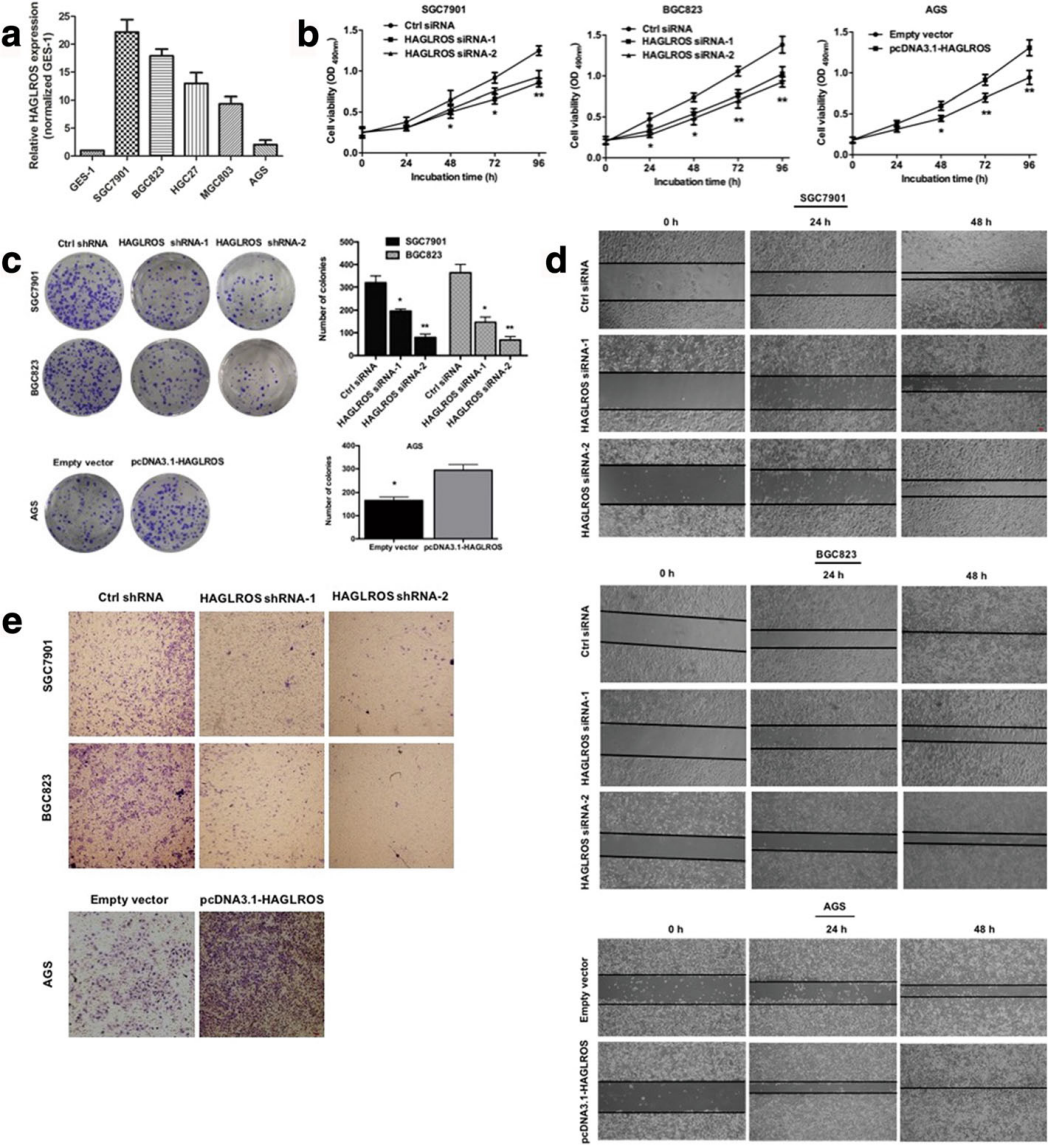
HAGLROS regulates GC cell proliferation and invasion in vitro. **a** Analysis of HAGLROS expression levels in GC cell lines compared with GES-1 cells by qRT-PCR. **b** Cell proliferation was determined by MTT assay after SGC-7901 and BGC-823 cells were transfected with siRNAs against HAGLROS and AGS cells were transfected with HAGLROS plasmid. **c** The representative results of colony formation assays using SGC-7901 and BGC-823 cells transfected with siRNAs against HAGLROS and AGS cells transfected with HAGLROS plasmid. **d** Cell migration was monitored by wound scratch assay; cell lines were treated the same as in (**b**) and (**c**). **e** Cell invasion was measured by Transwell assay; cell lines were treated the same as in (**b**) and (**c**)

HAGLROS expression was significantly higher in GC cell lines, including SGC-7901, BGC-823, HGC-27, MGC-803 and AGS, than in the normal gastric epithelium cell line GES-1 (Fig. 2a). To determine the function of increased HAGLROS expression in GC, we studied the effects of HAGLROS knockdown and overexpression on GC cell lines. The results showed that HAGLROS knockdown by siRNAs inhibited cell viability in BGC-823 and SGC-7901 cells, which have higher HAGLROS expression. In contrast, HAGLROS overexpression by cDNA transfection increased cell viability in AGS cells, which have lower HAGLROS expression (Fig. 2b). HAGLROS knockdown decreased clone formation in BGC-823 and SGC-7901 cells, while HAGLROS overexpression promoted clone formation in AGS cells (Fig. 2c). In the wound scratch assay, HAGLROS knockdown decreased wound healing in both BGC-823 and SGC-7901 cells, and HAGLROS overexpression increased wound healing in AGS cells (Fig. 2d and Additional file 5: Figure S2a). HAGLROS knockdown decreased cell invasion in both BGC-823 and SGC-7901 cells, while HAGLROS overexpression strengthened invasion in AGS cells (Fig. 2e). Altogether, these findings suggested that increased HAGLROS expression in GC contributed to GC cell proliferation and migration. Transcription efficiencies of knockdown and overexpression were shown in the Additional file 5: Figure S2b.

### HAGLROS promotes GC cell tumorigenesis in vivo

**Fig. 3:**
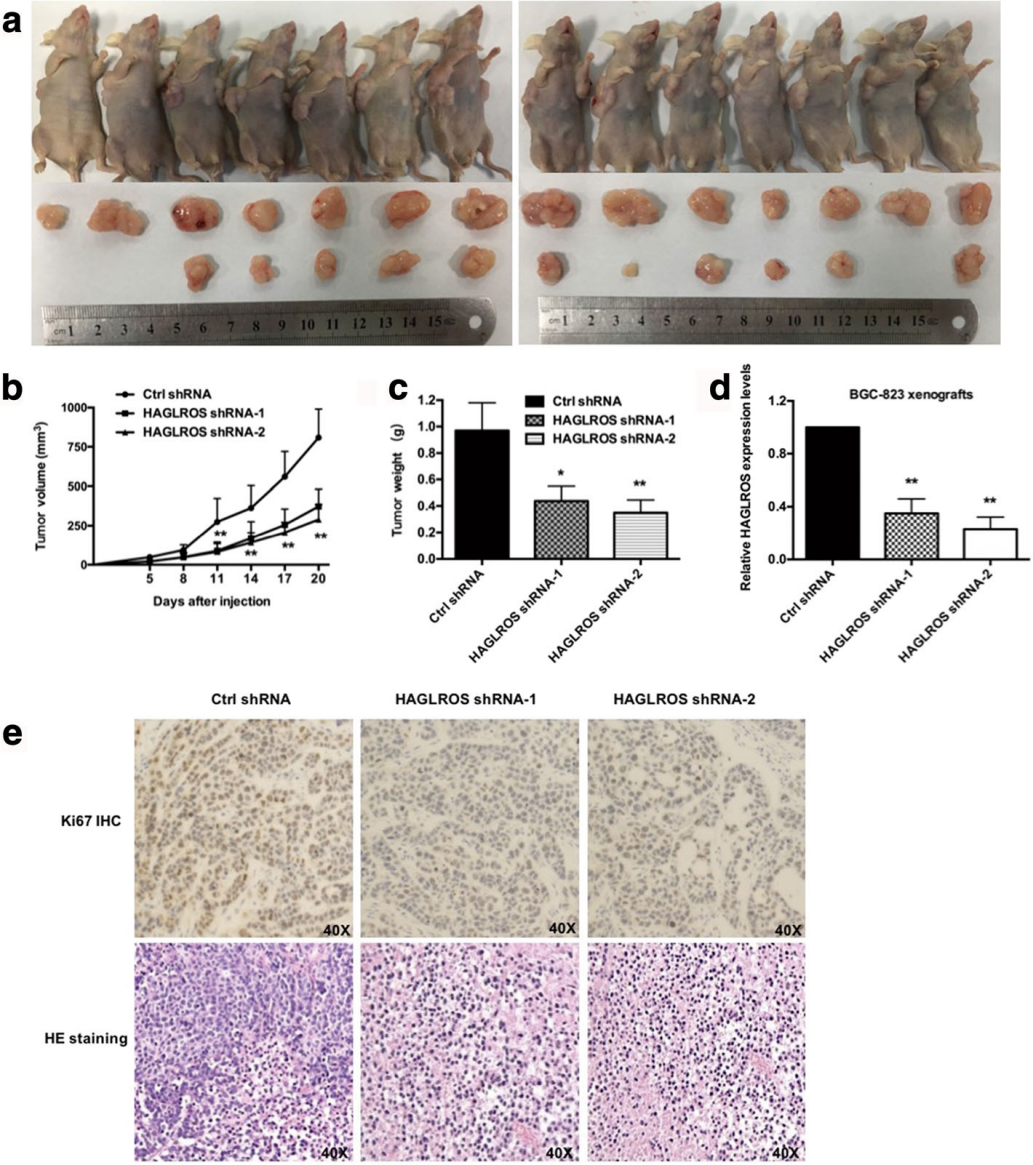
HAGLROS promotes GC cell tumorigenesis in vivo. **a** BGC-823 cells transfected with Ctrl shRNA and HAGLROS shRNA were injected respectively into nude mice (*n* = 7), which were killed by carbon dioxide euthanasia 20 days after injection. **b** Tumor volumes were calculated every 3 days beginning 5 days after injection. Bars indicate SD. **c** Tumor weights were represented as the means of tumor weights ± SD. **d** Transfection efficiency was tested by qRT-PCR. **e** The tumor sections underwent IHC staining using antibodies against Ki-67 and HE staining. Error bars indicate means ± S.E.M. *P < 0.05, **P < 0.01

In a nude mice xenograft model, SGC7901 cells with recombinant adenoviral vector producing shRNA against HAGLROS were inoculated into the flanks of mice, and SGC7901 cells infected with adenoviral vector carrying control shRNA were inoculated into the opposite flank of the same mouse as a control. Ad-shRNAs significantly inhibited tumorigenesis in vivo, as tumor weight and size were obviously decreased compared with the controls (Fig. 3a-c). We considered shRNAs to be appropriate for HAGLROS knockdown until the subcutaneous tumors were harvested and tested for relative HAGLROS expression levels (Fig. 3d). Furthermore, we detected stronger Ki-67 expression in tumors derived from control shRNA expression than those derived from HAGLROS shRNA expression, and HE staining showed similar changes (Fig. 3e).

### STAT3 induces HAGLROS expression by functioning as a transcription factor

**Fig. 4:**
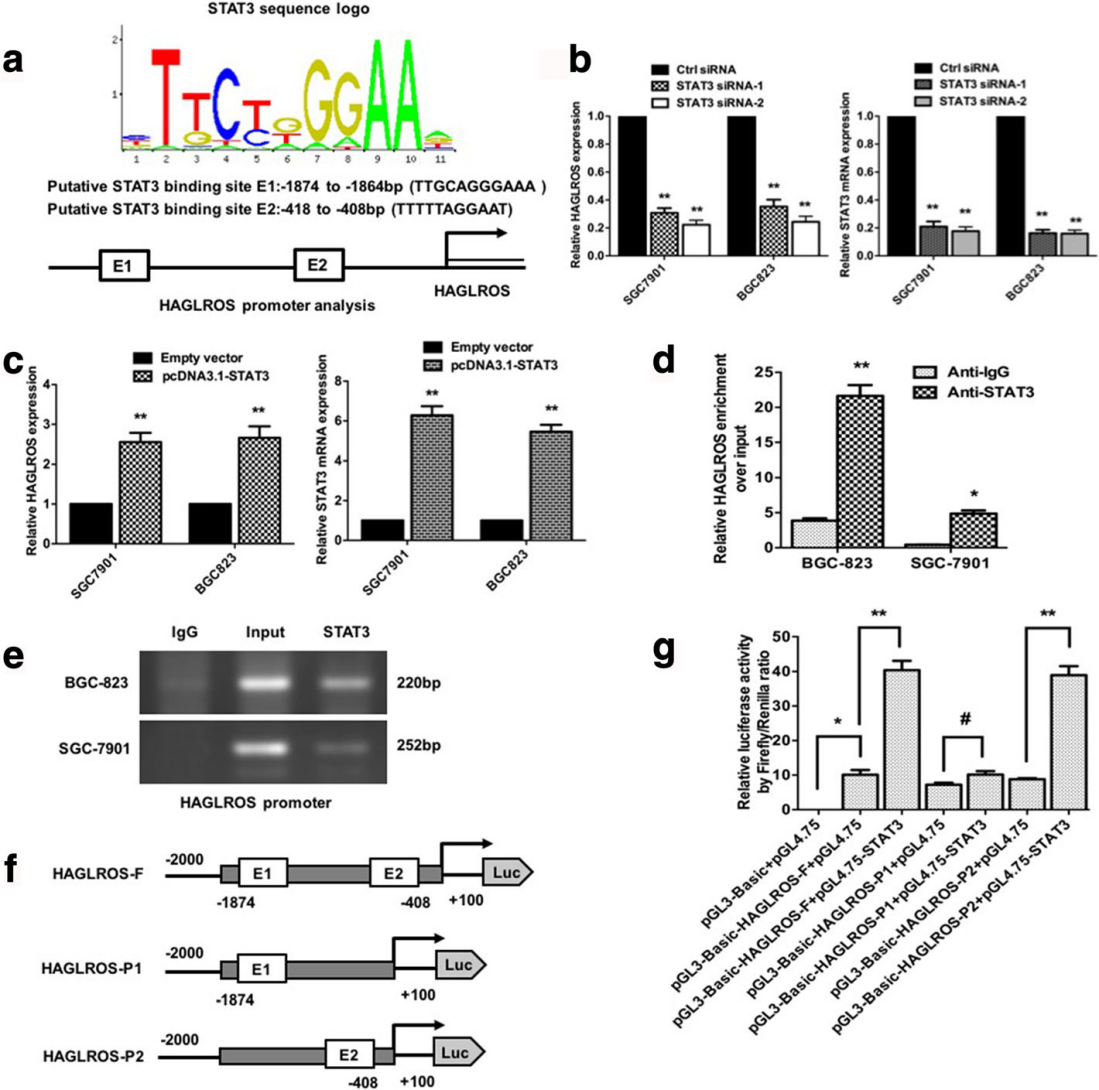
STAT3 induces HAGLROS expression as a transcription factor. **a** The predicted sites of STAT3-binding in human HAGLROS promoter by gene sequence analysis. **b** The effect of STAT3 knockdown on HAGLROS expression and the transfection efficiency of STAT3 siRNAs. **c** The effect of STAT3 overexpression on HAGLROS expression and the transfection efficiency of STAT3 plasmid. **d** Quantitative ChIP assays were used to show direct binding of STAT3 to endogenous HAGLROS promoter regions. **e** The representative blot of the binding of STAT3 to endogenous HAGLROS promoter regions. **f** and **g** A dual-luciferase reporter assay was performed by co-transfecting the full-length HAGLROS promoter (HAGLROS-F) or deleted HAGLROS E1 or E2 fragment (HAGLROS-P2 and HAGLROS-P1) with STAT3 plasmid or empty vectors in 293T cells. Error bars indicate the means ± S.E.M. *P < 0.05, **P < 0.01, # *P* > 0.05

The promoter region of HAGLROS was identified by the public website (http://www.ensembl.org/index.html) and we focused on the first 2000 bp upstream the transcription start site. We performed a computational screen based on computer algorithms (Jaspar: http://jaspar.genereg.net/) and found that 125 putative sites with scores > 10 were predicted in the 2000 bp upstream of HAGLROS transcript. We measured 10 common transcriptional factors (TCF3, SP1, STAT3, SP3, KLF5, EN1, CDX1, STAT4, TCF12 and HOXB2) to detect HAGLROS expression upon knockdowns of potential transcriptional factors and found that only STAT3 had an obvious corresponding change. The putative E1 and E2 STAT3 binding sites located between −1874 to −1864 bp (TTGCAGGGAAA) and between −418 bp to −408 bp (TTTTTAGGAAT) of the HAGLROS promoter, respectively (Fig. 4a). To verify whether high expression of HAGLROS was mediated by STAT3, we overexpressed STAT3 by cDNA transfection and knocked down STAT3 using an siRNA targeting STAT3. Upon STAT3 down-regulation, HAGLROS expression was markedly decreased in BGC-823 and SGC-7901 cells (Fig. 4b), while upon STAT3 overexpression, HAGLROS expression was significantly increased in BGC-823 and SGC-7901 cells (Fig. 4c). Chromatin immunoprecipitation (ChIP) assay was used to validate whether STAT3 could bind to the predicted site on the HAGLROS promoter region. The results showed that HAGLROS enrichment over input markedly increased STAT3 antibody levels compared to IgG antibody levels in BGC-823 and SGC-7901 cells (Fig. 4d and e).

To investigate whether STAT3 directly transcriptionally regulates HAGLROS expression, we cloned the full promoter region of HAGLROS and E1 or E2 promoter region mutants into luciferase reporter plasmids (Fig. 4f). As shown in Fig. 4g, luciferase assays showed that STAT3 induced the promoter activity of HAGLROS in 293T cells transfected with the full promoter region of HAGLROS. After transfection of the STAT3 expression plasmid, the P1 mutant (not containing the E2 STAT3 binding site) caused a significant reduction in promoter activity compared to the full-length promoter construct, but this reduction was not seen in the P2 mutant (not containing the E1 STAT3 binding site). These results indicate that the STAT3 binding site in the promoter region of HAGLROS is located the E2 binding site (−418 bp to −408 bp) instead of the E1 binding site (−1874 bp to −1864 bp).

### HAGLROS, as a mainly cytoplasmic lncRNA, serves as a sponge for miR-100-5p

**Fig. 5:**
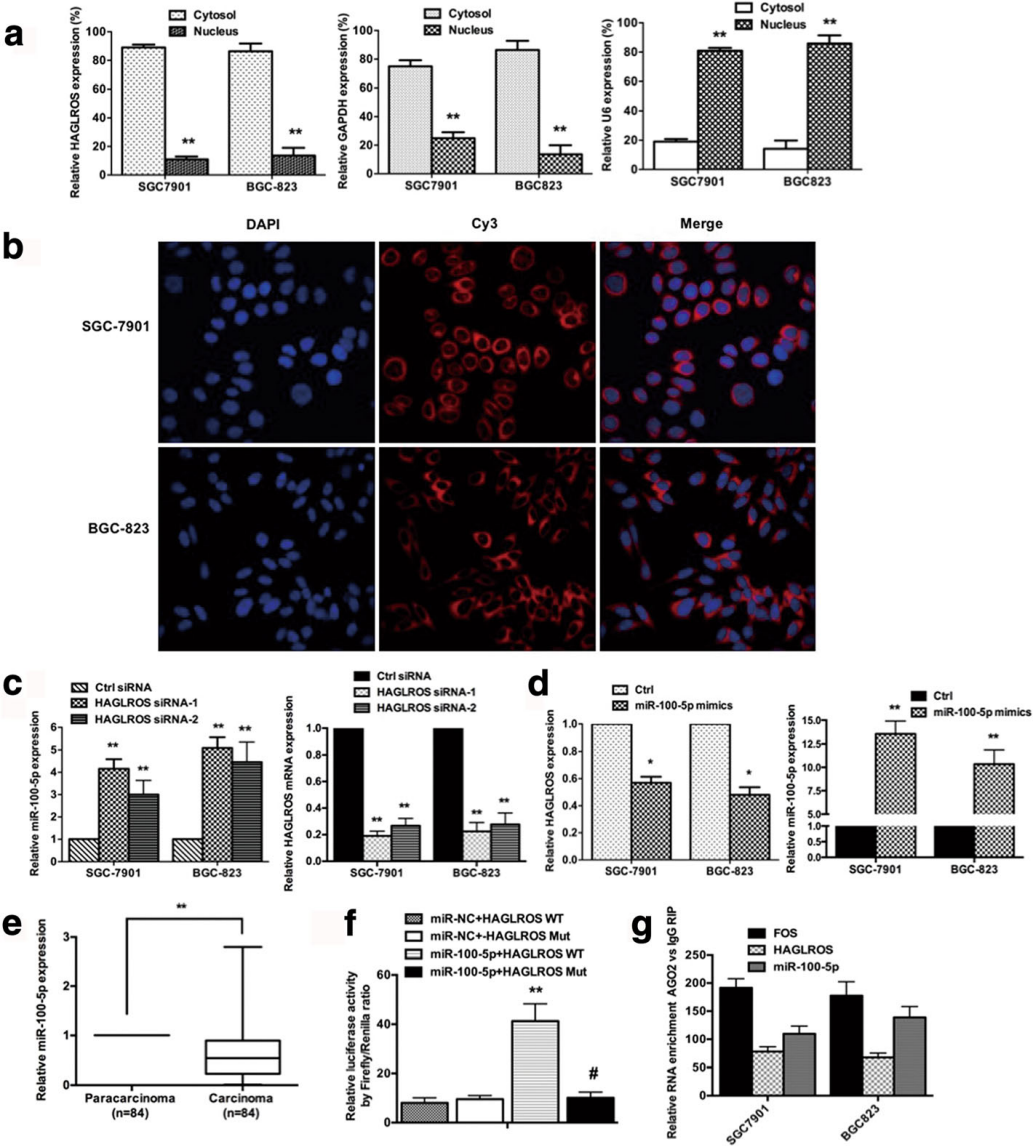
Subcellular localization of HAGLROS and its “sponge” function as a ceRNA competing with miR-100-5p. **a** RNA was extracted from the nuclear and the cytoplasmic fractions of SGC-7901 and BGC-823 cells and HAGLROS expression of the nuclear and the cytoplasmic fraction was measured by qRT-PCR. GAPDH was used as a cytosolic marker, and U6 was used as a nuclear marker. **b** FISH was used to confirm HAGLROS location in SGC-7901 and BGC-823 cells, using Cy3 probes for HAGLROS, DAPI for nuclear staining. **c** miR-100-5p expression was examined in SGC-7901 and BGC-823 cells with HAGLROS knockdown by siRNAs, and HAGLROS expression was tested to determine the transfection efficiencies. **d** HAGLROS levels were examined in SGC-7901 and BGC-823 cells transfected with miR-100-5p, and miR-100-5p levels were tested for transfection efficiencies. **e** The expression of miR-100-5p in tumor samples of GC compared to adjacent non-cancerous tissues. **f** Wild-type or mutant HAGLROS plasmid was co-transfected with miR-NC or miR-100-5p mimics into 293T cells, and relative luciferase activities were measured to determine the level of interaction between miR-100-5p and HAGLROS. **g** RNA levels in immunoprecipitates are presented as fold enrichment relative to IgG in AGO_2_ cells by RIP experiment. Error bars indicate the means ± S.E.M. *P < 0.05, **P < 0.01, #*P* < 0.05

To investigate the mechanism by which HAGLROS contributed to the malignant phenotypes of GC cells, we studied the localization of HAGLROS based on its activity within different subcellular areas. After GC cells were partitioned into nuclear and cytoplasmic fractions, we found that HAGLROS was localized preferentially to the cytoplasm (Fig. 5a). FISH experiments also showed that HAGLROS was mostly located in the cytoplasm (Fig. 5b) and further confirmed that HAGLROS functioned as a mainly cytoplasmic lncRNA. It is well known that cytoplasmic lncRNAs can bind directly to miRNA and function as sponges or compete with ceRNAs to control the availability of miRNA for binding to their target mRNAs [26, 27]. Bioinformatics analysis using miRcode (http://www.mircode.org/) and StarBase v2.0 (http://starbase.sysu.edu.cn/mirLncRNA.php) software suggested that HAGLROS could bind both miR-100-5p and the Argonaute 2 (Ago2) protein. Based on this prediction, we speculated that HAGLROS might act as a sponge in GC. In the GC cells, HAGLROS knockdown by siRNAs caused miR-100-5p up-regulation (Fig. 5c), while overexpression of miR-100-5p by transfection with mimics caused HAGLROS down-regulation (Fig. 5d). These results show that HAGLROS and miR-100-5p are competitively expressed. Moreover, the expression of miR-100-5p and HAGLROS showed an inverse correlation in tumor samples in the higher expression of HAGLROS in GC compared to the adjacent non-cancerous tissue (Fig. 5e).

To further determine the interaction of miR-100-5p and HAGLROS, we constructed luciferase vectors of wild-type and mutant HAGLROS (the binding site for miR-100-5p was mutated). Using a dual-luciferase reporter assay, we found that 293T cells transfected with wild-type HAGLROS and miR-100-5p mimics, but not the mutant HAGLROS, presented significantly decreased luciferase activity (Fig. 5f). We performed anti-AGO2 RIP to detect whether HAGLROS was regulated by miR-100-5p in an AGO2-dependent manner. Endogenous HAGLROS pull-down by AGO2 was significantly enriched in miR-100-5p-overexpressing cells (Fig. 5g).

### HAGLROS functions as a ceRNA to antagonize miR-100-5p-mediated mTOR mRNA degradation

**Fig. 6:**
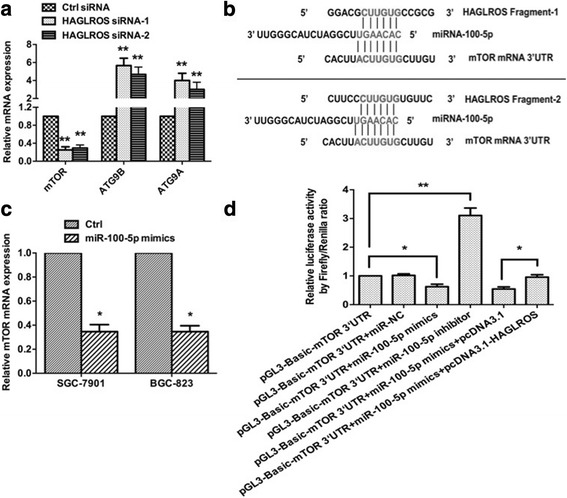
HAGLROS antagonized miR-100-5p-mediated mTOR mRNA degradation. **a** The relative expression levels of autophagy-related signals mTOR and ATG9A/9B were validated by qRT-PCR upon HAGLROS knockdown in accordance with RNA high-throughput sequencing guidelines. **b** Bioinformatic analysis of the interactions of HAGLROS with miR-100-5p and mTOR mRNA. **c** The effect of miR-100-5p overexpression by transfection with miR-100-5p mimics on mTOR mRNA level in GC cells. **d** Relative luciferase activity of mTOR mRNA 3’-UTR was determined after transfection with miR-100-5p mimics, miR-100-5p inhibitor or HAGLROS plasmid. Error bars indicate the means ± S.E.M. *P < 0.05, **P < 0.01

To identify potential functional targets of HAGLROS, we performed transcript RNA high-throughput sequencing. As shown in Additional file 6: Figure S3a, many genes involved in cell proliferation and autophagy were affected by HAGLROS. We confirmed expressions of a panel of genes which were up- and down-regulated by HAGLROS knockdown using qRT-PCR assays (Additional file 7: Figure S4). Furthermore, GO-term and pathway enrichment analysis showed that the differentially expressed genes about autophagy indeed enriched at a significant level by HAGLROS silencing (Additional file 6: Figure S3b and c). Of all the identified genes, mTOR and ATG9A/9B (Fig. 6a) are known to be autophagy-related signals. Bioinformatics analysis using miRcode (http://www.mircode.org/) and StarBase v2.0 (http://starbase.sysu.edu.cn/mirLncRNA.php) software suggested that HAGLROS could bind both miR-100-5p and mTOR mRNA (Fig. 6b). The fact that transfection of miR-100-5p mimics attenuated mTOR mRNA levels (Fig. 6c) suggested that mTOR might be the target of miR-100-5p. To verifying this hypothesis, the 3′-UTR of mTOR was cloned into a luciferase vector and transfected into 293T cells together with miR-100-5p mimics, an miR-100-5p inhibitor or a negative control. The miR-100-5p mimics significantly reduced the luciferase activity, while the miR-100-5p inhibitor markedly strengthened it, indicating that mTOR was a direct target of miR-100-5p. Subsequently, the 3′-UTR of mTOR was co-transfected with the HAGLROS plasmid and miR-100-5p mimics. HAGLROS overexpression negated the decrease in luciferase activity induced by overexpressing miR-100-5p (Fig. 6d). This result implied that HAGLROS bound to miR-100-5p and released mTOR from miR-100-5p, further demonstrating the existence of HAGLROS-mTOR crosstalk through competition for miR-100-5p binding.

### HAGLROS interacted with mTORC1 through binding with components of the complex

**Fig. 7:**
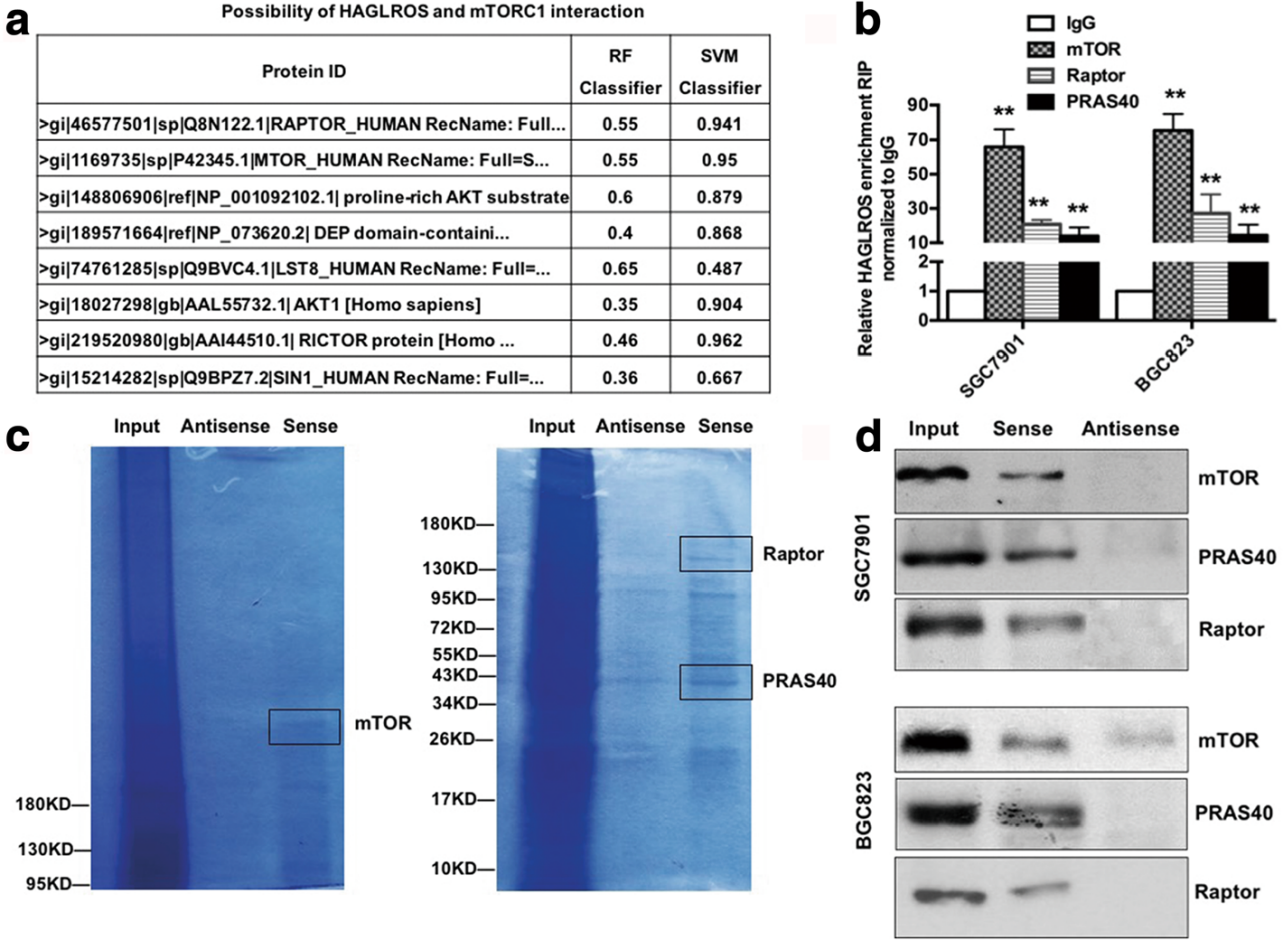
Bioinformatics prediction and experimental verification of HAGLROS binding to mTORC1 components. **a** Bioinformatic analysis of HAGLROS interacting with mTORC1 components. Values greater than 0.5 for both the RF classifier and the SVM classifier indicate a high possibility of interaction. **b** HAGLROS levels in immunoprecipitates are presented as fold enrichment in mTOR, Raptor and PRAS40 antibodies relative to IgG immunoprecipitates by RIP experiments. **c** Coomassie brilliant blue staining of mTOR, Raptor and PRAS40 protein levels by SDS-PAGE of immunoprecipitates from HAGLROS pulldown. **d** Western blot analysis of mTOR, Raptor and PRAS40 protein levels in immunoprecipitates from HAGLROS pulldown. **P < 0.01

To verify crosstalk of the cytoplasmic lncRNA HAGLROS and mTOR, we performed bioinformatics analysis using a web of RNA and protein interactions (http://pridb.gdcb.iastate.edu/RPISeq/) and found that the mTORC1 components mTOR, Raptor and PRAS40 could possibly interact with HAGLROS. In our analysis, both the RF classifier and the SVM classifier were larger than 0.5 (Fig. 7a). RIP experiments examined and confirmed the interaction of HAGLROS directly with mTOR, Raptor and PRAS40 in GC cells (Fig. 7b). Moreover, an RNA pulldown assay further identified that HAGLROS indeed bound with mTOR, Raptor and PRAS40 in GC cells (Fig. 7c and d).

### HAGLROS promotes GC progression through mTOR-mediated autophagy inhibition

**Fig. 8:**
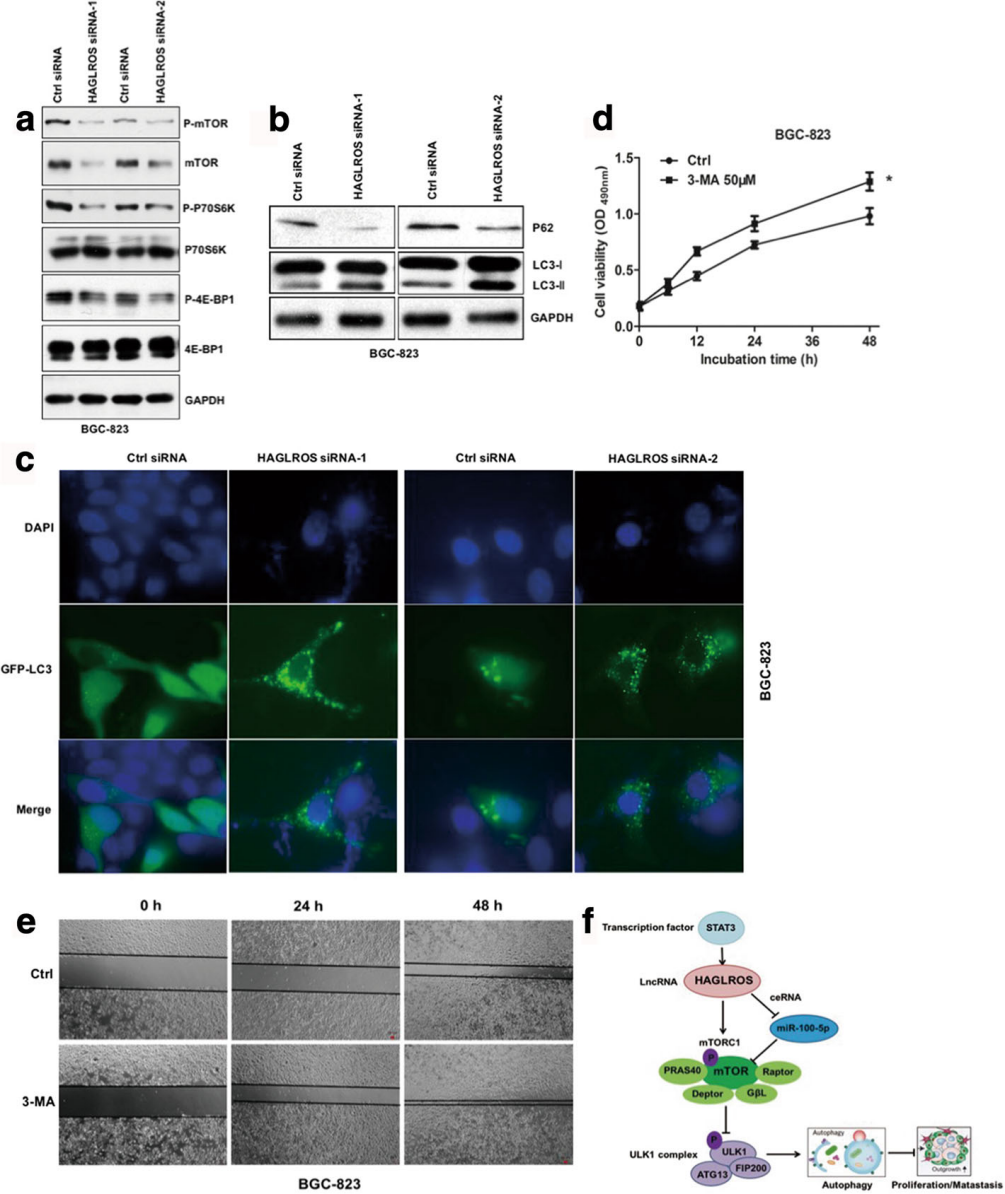
HAGLROS promotes GC progression through mTOR-mediated autophagy inhibition. **a** Upon knockdown of HAGLROS by siRNAs, mTORC1 activity, including phosphorylation of both mTOR and downstream molecules, was analyzed in BGC-823 cell lines by Western blot. **b** Autophagy markers P62 and LC3 were measured by Western blot in BGC-823 cell lines with HAGLROS knockdown. **c** LC3-II punctuation was determined by BGC-823 cells co-transfected with GFP-LC3 plasmid and HAGLROS siRNAs by immunofluorescence assays. **d** and (**e**) BGC-823 cells were treated with the autophagic inhibitor 3-methyladenine (3-MA), and cell viability and migratory ability were measured by MTT assay (**d**) and (**e**) wound scratch assay. **f** Summary of the molecular mechanisms of HAGLROS in gastric cancer cells. *P < 0.05

Usually, mTOR mediates signaling from its effectors, maintaining normal cell function and homeostasis. However, in various diseases, especially in cancer, this capacity is lost because of mutations or activation of signals upstream of mTOR that lead to persistent proliferation and tumor growth [28]. It is well known that mTORC1 inhibits autophagy through its interaction with autophagy-related genes. Western blot analysis showed that knockdown of HAGLROS markedly down-regulated mTORC1 activity and alleviated the phosphorylation of both mTOR and downstream molecules in BGC-823 cell lines (Fig. 8a). Upon HAGLROS knockdown, the autophagy markers LC3 and P62 underwent respective changes: the LC3-I to LC3-II transition increased, and P62 levels decreased (Fig. 8b). Immunofluorescence assay demonstrated that LC3-II punctuation was markedly elevated in BGC-823 cells transfected with HAGLROS siRNAs compared to Ctrl siRNA (Fig. 8c). Furthermore, we found increasing mTOR pathway members in HAGLROS silenced cells inhibited the autophagic phenotype (Additional file 8: Figure S5a). All these findings suggested that HAGLROS could inhibit autophagy through activating mTORC1 signals, at least in part. Therefore, we presumed that HAGLROS contributed to GC development by inhibiting autophagy, and the next investigation confirmed this hypothesis. We treated BGC-823 cells with the autophagic inhibitor 3-methyladenine (3-MA) and found that cell viability and migratory ability were obviously increased (Fig. 8d, e and Additional file 8: Figure S5b). These results validate that in GC cells, inhibition of autophagy promotes tumor development, at least in part. Taken together, these results indicate that HAGLROS contributes to GC proliferation and migration by mTORC1-mediated autophagy inhibition.

## Discussion

Most cancers are genetic diseases that change the flow of cellular information and thus disrupt cellular homeostasis and promote proliferation. More and more evidence confirms that lncRNAs have important roles in GC carcinogenesis, proliferation and metastasis through survival signaling pathways [29]. Differential display analysis of human prostate cancers identified Differential display 3, also known as PCA3, this lncRNA was approved by the Food and Drug Administration (FDA) for prostate cancer diagnosis and was the first case of a lncRNA being used for clinical testing by FDA approval [30, 31]. Similarly, analyses of gastric secretions from patients with GC identified lncRNA-AA174084 as a biomarker capable of differentiating between GC and benign disorders of the gastric epithelium [32]. Many lncRNAs have been reported to be associated with GC, but the most characteristic biomarker remains unclear. In the present study, we found that the lncRNA HAGLROS was significantly overexpressed in GC compared to corresponding non-tumor tissues. The high expression levels of HAGLROS in GC were positively correlated with invasion depth and TNM stage. In addition, high HAGLROS expression in GC tissues was associated with a poor prognosis and could be an independent prognostic indicator. These results suggested that HAGLROS might have important roles in GC progression.

The dysregulation of lncRNAs influences various pathological processes. Alike protein-coding transcripts, the transcription of lncRNAs is subject to typical epigenetic-mediated and transcription factor-mediated regulation. STAT3 is both a transcriptional activator and an oncogene under normal physiological conditions. However, much evidence indicates that STAT3 is constitutively activated in cancers, playing a crucial role in tumor onset and progression. In addition to its traditional role in cancer cell proliferation, invasion, and migration, STAT3, as a transcription factor, promotes cancer development by altering the expression of other genes in cancer cells [33]. Furthermore, overexpression of STAT3 has been observed in various types of tumors, including GC. In this study, we found that HAGLROS was a direct target of transcription factor STAT3, which was affirmed by STAT3 binding to the predicted site of the promoter region of HAGLROS and by STAT3 causing significant induction of HAGLROS promoter activity, as determined by luciferase reporter assay (Fig. 4). Therefore, up-regulation of HAGLROS in GC is partly due to STAT3 activation during tumor progression.

lncRNA regulation of cellular processes depends in part on lncRNA cellular localization: nuclear lncRNAs are enriched for functionality involving chromatin interactions, transcriptional regulation, and RNA processing, while cytoplasmic lncRNAs can modulate mRNA stability or translation and influence cellular signaling cascades [1]. LncRNA modulation of RNA metabolism is an emerging theme for lncRNAs that are enriched in the cytoplasm, where the lncRNAs participate in cellular biological processes by functioning as ceRNAs or “RNA sponges” regulating mRNA stability, mRNA alternative splicing, and protein localization [34]. In the present study, we report that HAGLROS is localized preferentially in the cytoplasm, as determined by subcellular fractionation and FISH experiments. HAGLROS functions as a ceRNA to antagonize miR-100-5p-mediated mTOR mRNA degradation; HAGLROS and mTOR interact through competition for miR-100-5p binding. In addition, we observed that HAGLROS, as a mainly cytoplasmic lncRNA, interacts directly with mTORC1 components (mTOR, Raptor and PRAS40) and activated the mTORC1 pathway by stabilizing the complex’ structure. In accordance with the above statements, cytoplasmic lncRNA HAGLROS has two mechanisms to activate the mTOR pathway and thus inhibit autophagy. On the one hand, HAGLROS functions as a ceRNA to increase mTOR mRNA expression through competing with miR-100-5p, on the other hand, HAGLROS binds mTORC1 key proteins to activate the complex and finally participates in cellular biological processes. mTORC1, a master positive regulator of cell growth and proliferation, forms the integrational hub of an extensive network of regulatory proteins that transmit extrinsic and intrinsic signals regarding cellular nutritional status. The influence of mTORC1 on cellular metabolism is substantial considering its regulation by common oncogenic signaling pathways (e.g., PIK3CA-AKT1 and RAS-ERK) [35] and the observation that aberrant mTORC1 signaling is found in 40% to 90% of human cancers [36].

Given the intimate relationship between mTORC1 signaling and autophagy, it is likely that cancer-associated sequence changes in the mechanistic target of rapamycin or mTOR and/or aberrant mTOR protein expression would perturb autophagy, making autophagy an important mediator of the effects of this common dysregulation in human cancer [37]. Here, we monitored the knockdown of HAGLROS and its effects on autophagy through the mTOR pathway. The functional relevance of autophagy in tumor formation and progression remains controversial. Intriguingly, many oncogenes and tumor suppressor genes affect autophagic pathways, and the dysregulation of the autophagic process contributes to malignant transformation [38]. Many tumor suppressor proteins, such as p53, phosphatase and tensin homolog (PTEN) and death-associated protein kinase (DAPK), that provide constitutive input signals to activate autophagy are mutated in multiple cancers. Conversely, oncogenes, including Akt, mTOR and Bcl-2, inhibit autophagic processes indicating that elevated autophagy signaling may contribute to tumor suppression [39, 40]. In GC cells, HAGLROS and mTOR levels are consistently parallel, with increased levels inhibiting autophagy and promoting tumor proliferation and invasion. The regulatory mechanism of HAGLROS was summarized in the Fig. 8f.

## Conclusions

Our study demonstrates that the GC-associated lncRNA HAGLROS is an oncogenic lncRNA that promotes tumorigenesis and progression through mTOR pathway-mediated autophagy suppression by serving as a ceRNA for miR-100-5p and as a cytoplasmic scaffold to bind mTORC1. Our findings support the idea that lncRNAs such as HAGLROS play crucial roles in GC progression and prove that HAGLROS is a potential effective target for treating GC.

## Additional files

Additional file 1: Table S1. The relationship between HAGLROS expression and clinicopathological factors of GC patients. (DOCX 14 kb)

Additional file 2: Table S2. Primers used for qRT-PCR,RT-PCR, CHIP and siRNAs/shRNA oligonucleotides. (XLSX 12 kb)

Additional file 3: Figure S1. The relative lncRNAs expression from 12 GC patients were validated by qRT-PCR. Error bars indicate the means ± S.E.M. *P < 0.05, **P < 0.01 for carcinoma vs paracarcinoma. (TIFF 165 kb)

Additional file 4: Table S3. Univariate and multivariate analyses of the clinicopathological factors for overall survival in 84 patients with GC. (DOCX 16 kb)

Additional file 5: Figure S2. (a) Relative areas of the wound scratch assay by Image J software. *P < 0.05 for siRNAs vs Ctrl siRNAs and pcDNA3.1-HAGLROS vs vector. (b) Transcription efficiencies of siRNAs, shRNAs and pcDNA3.1-HAGLROS. **P < 0.01. Error bars indicate the means ± S.E.M. (TIFF 1170 kb)

Additional file 6: Figure S3. RNA-sequencing analysis of HAGLROS siRNA vs Ctrl. (a) 194 differentially expressed genes upon HAGLROS siRNA vs Ctrl. (b) GO analysis of differentially expressed genes. (c) Pathway enrichment analysis of differentially expressed genes. (TIFF 3460 kb)

Additional file 7: Figure S4. The relative expression levels of downstream signals were validated by qRT-PCR upon HAGLROS knockdown in accordance with RNA high-throughput sequencing guidelines. (a) Down-regulated genes were validated by qRT-PCR upon HAGLROS knockdown. (b) Up-regulated genes were validated by qRT-PCR upon HAGLROS knockdown. Error bars indicate the means ± S.E.M. *P < 0.05, **P < 0.01. (TIFF 1840 kb)

Additional file 8: Figure S5. (a) Increasing mTOR pathway members in HAGLROS silenced cells inhibited the autophagic phenotype. (b) Relative areas of the wound scratch assay by Image J software, corresponding Fig. 8e. (TIFF 1880 kb)

## Abbreviations

3-MA: 3-methyladenine
Ago2: Argonaute 2
ceRNA: Competing endogenous RNA
ChIP: Chromatin immunoprecipitation
DAPK: Death-associated protein kinase
DEPTOR: DEP domain containing mTOR-interacting protein
FDA: Food and Drug Administration
GC: Gastric cancer
GEO: Gene Expression Omnibus
IHC: Immunohistochemical
lncRNAs: Long noncoding RNAs
mLST8: Mammalian lethal with sec-13 protein 8
mSin1: Mammalian stress-activated map kinase-interacting protein 1
mTOR: Mammalian target of rapamycin
mTORC1: mTOR complex 1
mTORC2: mTOR complex 1
NCBI: National Center for Biotechnology Information
ncRNAs: Noncoding RNAs
PRAS40: Proline-rich Akt substrate 40 kDa
protor1/2: Protein observed with rictor 1 and 2
PTEN: Phosphatase and tensin homolog
Raptor: Regulatory-associated protein of mammalian target of rapamycin
Rictor: Rapamycin-insensitive companion of mTOR
RIP: RNA immunoprecipitation
TCGA: The Cancer Genome Atlas
