# Supplementary material for: STAT3-induced lncRNA HAGLROS overexpression contributes to the malignant progression of gastric cancer cells via mTOR signal-mediated inhibition of autophagy
Source: Mol Cancer. 2018 Jan 12;17:6. doi: 10.1186/s12943-017-0756-y (PMC5767073; doi:10.1186/s12943-017-0756-y)
Supplement: Supplementary file 1 — The relationship between HAGLROS expression and clinicopathological factors of GC patients. (DOCX 14 kb) [file 12943_2017_756_MOESM1_ESM.docx]

Table S1. The relationship between HAGLROS expression and clinicopathological factors of GC patients

| Clinical parameters | HAGLROS levels | P-value^a^ |
| --- | --- | --- |
|  | High (n=54) Low (n=30) |  |
| Age  ≤ 60  >60  Gender  Male  Female  Histologic differentiation  Low or undifferentiation  Middle or high  Invasion depth  T1  T2 or above  TNM Stages  I/II  III/IV  Lymphatic metastasis  Yes  No  Distant metastasis  Yes  No | 20 14  34 16  30 20  24 10  31 12  23 18  8 12  46 18  22 22  32 8  32 12  22 18  9 1  45 29 | 0.389  0.320  0.126  0.009**  0.004**  0.090  0.071 |

^a^ Chi-squared test; ***P*<0.01.
