# Supplementary figures and images for: STAT3-induced lncRNA HAGLROS overexpression contributes to the malignant progression of gastric cancer cells via mTOR signal-mediated inhibition of autophagy
Source: Mol Cancer. 2018 Jan 12;17:6. doi: 10.1186/s12943-017-0756-y (PMC5767073; doi:10.1186/s12943-017-0756-y)

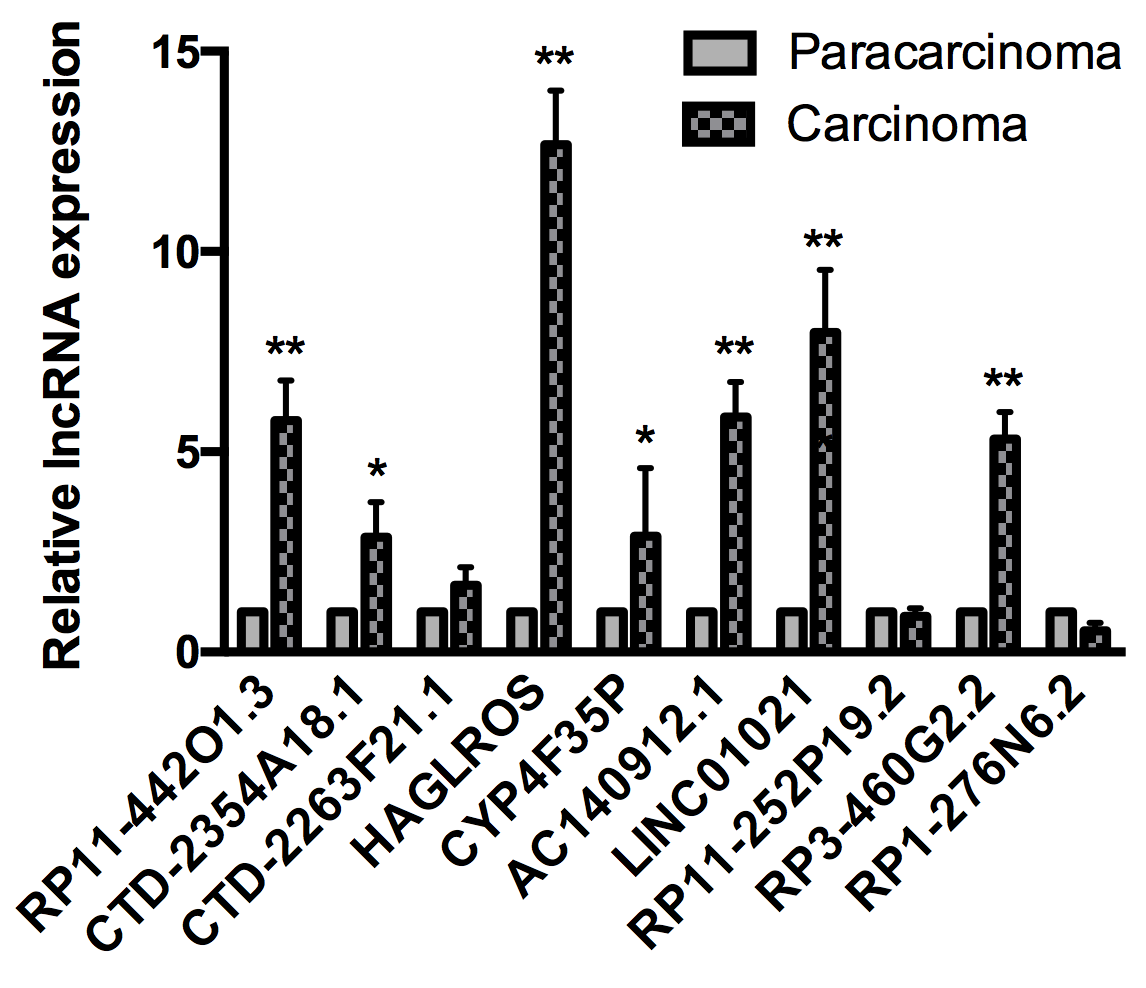

Supplement: Supplementary file 3 — The relative lncRNAs expression from 12 GC patients were validated by qRT-PCR. Error bars indicate the means ± S.E.M. *P < 0.05, **P < 0.01 for carcinoma vs paracarcinoma. (TIFF 165 kb) [file 12943_2017_756_MOESM3_ESM.tiff]

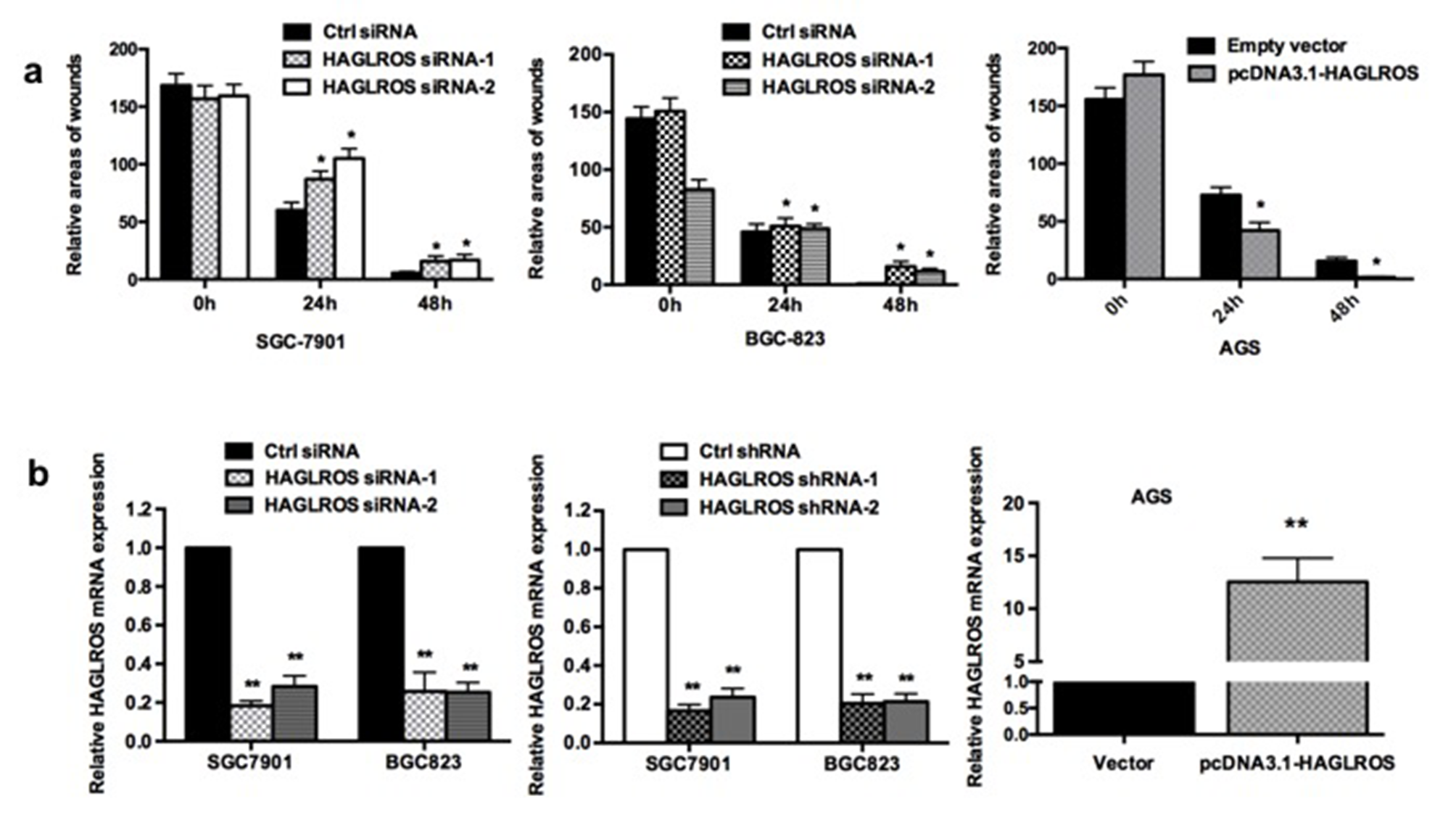

Supplement: Supplementary file 5 — (a) Relative areas of the wound scratch assay by Image J software. *P < 0.05 for siRNAs vs Ctrl siRNAs and pcDNA3.1-HAGLROS vs vector. (b) Transcription efficiencies of siRNAs, shRNAs and pcDNA3.1-HAGLROS. **P < 0.01. Error bars indicate the means ± S.E.M. (TIFF 1170 kb) [file 12943_2017_756_MOESM5_ESM.tif]

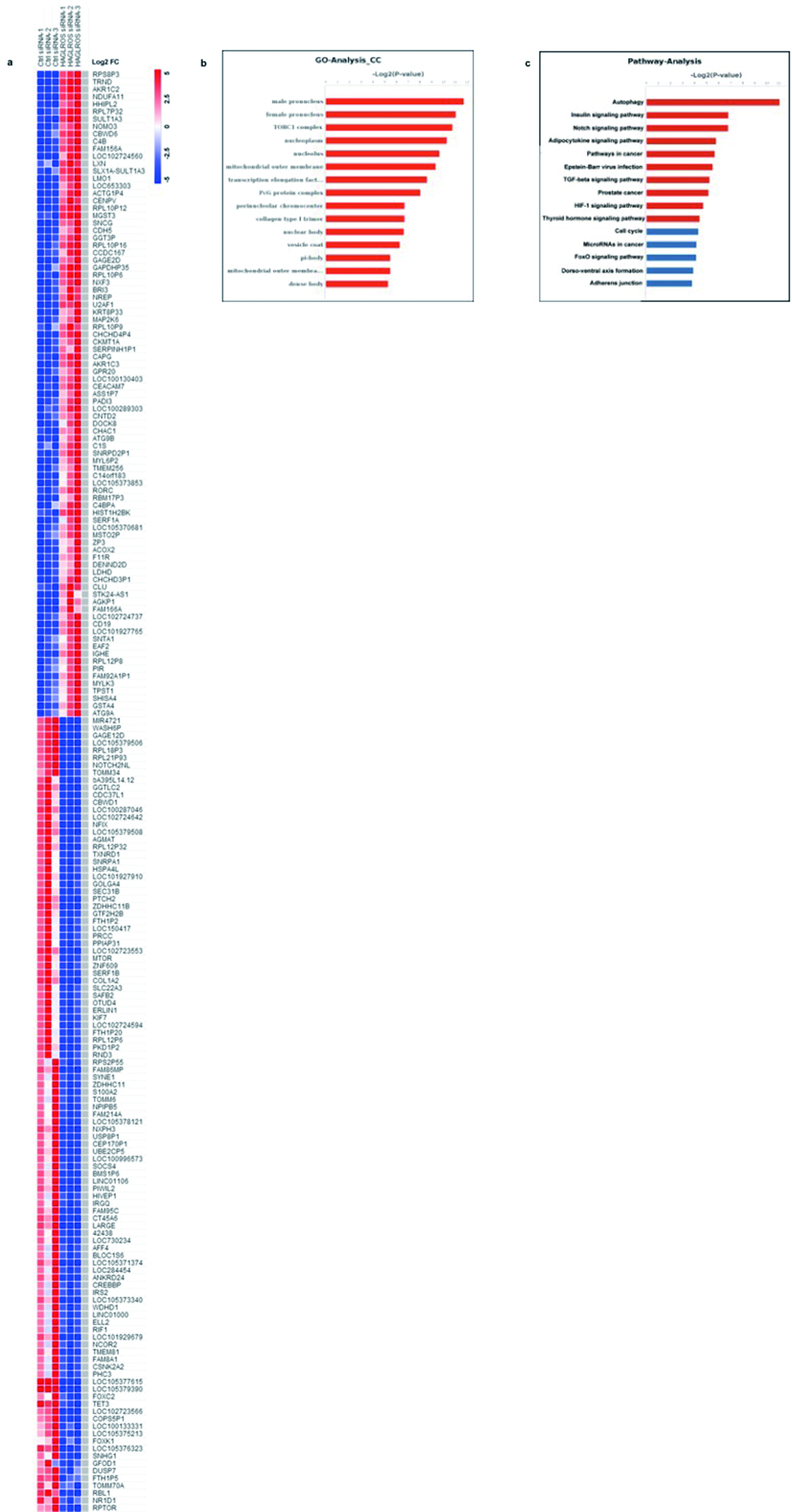

Supplement: Supplementary file 6 — RNA-sequencing analysis of HAGLROS siRNA vs Ctrl. (a) 194 differentially expressed genes upon HAGLROS siRNA vs Ctrl. (b) GO analysis of differentially expressed genes. (c) Pathway enrichment analysis of differentially expressed genes. (TIFF 3460 kb) [file 12943_2017_756_MOESM6_ESM.tif]

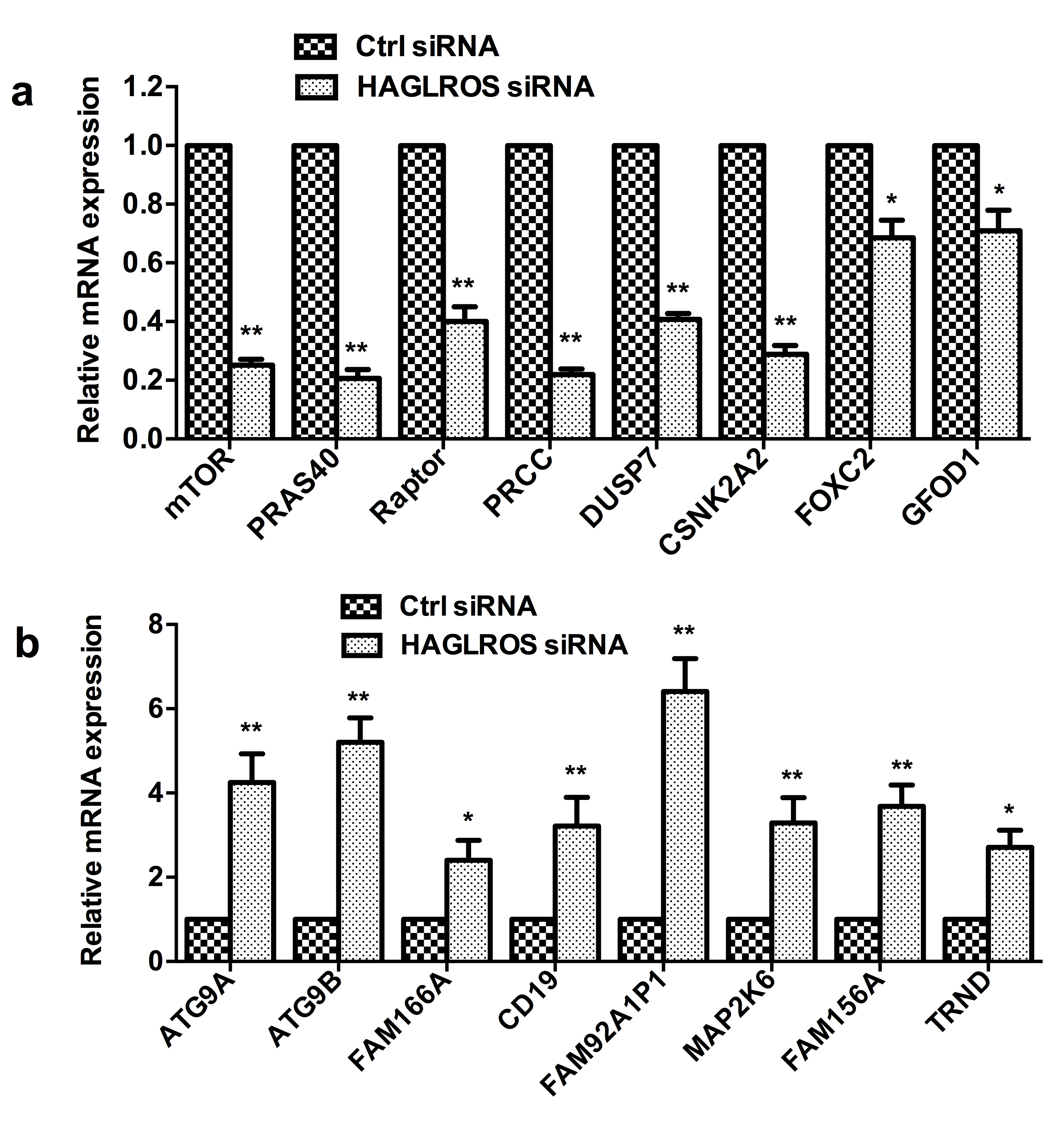

Supplement: Supplementary file 7 — The relative expression levels of downstream signals were validated by qRT-PCR upon HAGLROS knockdown in accordance with RNA high-throughput sequencing guidelines. (a) Down-regulated genes were validated by qRT-PCR upon HAGLROS knockdown. (b) Up-regulated genes were validated by qRT-PCR upon HAGLROS knockdown. Error bars indicate the means ± S.E.M. *P < 0.05, **P < 0.01. (TIFF 1840 kb) [file 12943_2017_756_MOESM7_ESM.tiff]

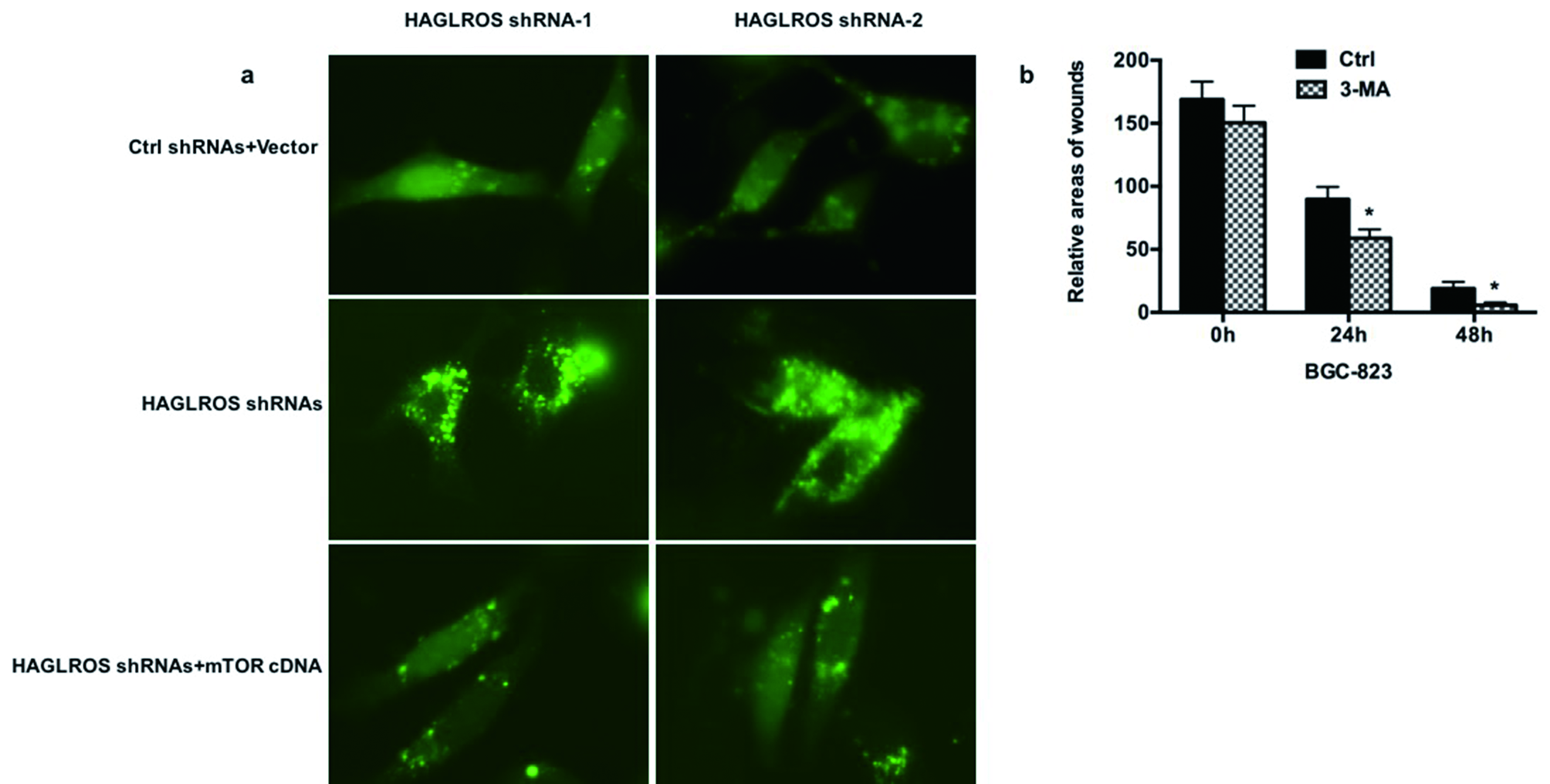

Supplement: Supplementary file 8 — (a) Increasing mTOR pathway members in HAGLROS silenced cells inhibited the autophagic phenotype. (b) Relative areas of the wound scratch assay by Image J software, corresponding Fig. 8e. (TIFF 1880 kb) [file 12943_2017_756_MOESM8_ESM.tif]
