# Supplementary material for: STAT3-induced lncRNA HAGLROS overexpression contributes to the malignant progression of gastric cancer cells via mTOR signal-mediated inhibition of autophagy
Source: Mol Cancer. 2018 Jan 12;17:6. doi: 10.1186/s12943-017-0756-y (PMC5767073; doi:10.1186/s12943-017-0756-y)
Supplement: Supplementary file 4 — Univariate and multivariate analyses of the clinicopathological factors for overall survival in 84 patients with GC. (DOCX 16 kb) [file 12943_2017_756_MOESM4_ESM.docx]

Table S3. Univariate and multivariate analyses of the clinicopathological factors for overall survival in 84 patients with GC

| Risk factors | DFS |
| --- | --- |
|  | HR 95% CI *P*-value |
| **Univariate analysis**  Age (≤60, >60)  Sex (Male, Female)  HAGLROS expression  Histological grade (Low or undiffer, Middle or high)  Lymph node metastasis (Yes or No)  Tumor invasion depth (T1, T2 or above)  TNM stage (I/II, III/IV)  Distant metastasis (Yes or No)  **Multivariate analysis**  HAGLROS expression  Lymph node metastasis (Yes or No)  Tumor invasion depth (T1, T2 or above)  TNM stage (I/II, III/IV) | 1.129 0.594-0.989 0.752  0.632 0.304-0.980 0.436  2.122 1.010-4.356 0.012*  1.003 0.502-2.238 0.317  0.408 0.240-0.778 0.003**  1.642 0.643-2.814 0.034*  3.628 2.021-8.349 0.001**  0.462 0.120-1.255 0.066  1.096 0.823-1.162 0.005**  2.387 1.221–4.416 0.001**  2.021 0.654–7.483 0.092  2.285 1.021–3.976 0.019* |

Abbreviations: DFS, disease-free survival; CI, confidence interval; HR, hazard

ratio. **P*<0.05; ***P*<0.01.
